# Molecular detection of tick-borne pathogens in cattle ticks from the Lao People’s Democratic Republic

**DOI:** 10.1186/s13071-025-07167-2

**Published:** 2025-12-06

**Authors:** Alongkorn Nonthapa, Rutchanee Rodpai, Tongjit Thanchomnang, Patcharaporn Boonroumkaew, Lakkhana Sadaow, Virasak Banouvong, Sakhone Laymanivong, Kanchana Thinnabut, Ubon Tangkawanit, David Blair, Pewpan M. Intapan, Wanchai Maleewong, Oranuch Sanpool

**Affiliations:** 1 Department of Parasitology, Faculty of Medicine, Khon Kaen University, Khon Kaen, Thailand; 2 Mekong Health Science Research Institute, Khon Kaen University, Khon Kaen, Thailand; 3 Faculty of Medicine, Mahasarakham University, Maha Sarakham, Thailand; 4 Centre of Malariology, Parasitology and Entomology, Ministry of Health, Vientiane, Lao People’s Democratic Republic; 5 Department of Entomology and Plant Pathology, Faculty of Agriculture, Khon Kaen University, Khon Kaen, Thailand; 6 College of Science and Engineering, James Cook University, Townsville, QLD 4811 Australia

**Keywords:** Tick-borne pathogens, Cattle ticks, Molecular survey, Lao People’s Democratic Republic

## Abstract

**Background:**

Tick-borne pathogens threaten livestock health and productivity in Southeast Asia. Despite growing regional interest, epidemiological data from the Lao People’s Democratic Republic (Lao PDR) remain insufficient and underreported.

**Methods:**

We collected 227 ticks from 63 cattle across northern, central, and southern Lao PDR. Species were morphologically identified as *Rhipicephalus microplus* and *Rhipicephalus sanguineus* sensu lato and confirmed by sequencing of the cytochrome c oxidase subunit I gene. Pathogen screening was conducted via polymerase chain reaction (PCR), with sequence identities verified using the GenBank database. Multivariate analyses assessed regional variation.

**Results:**

Detected pathogens included the apicomplexan protozoans *Babesia bigemina* (7%), *Babesia bovis* (2.6%), and *Theileria* sp. (10.6%) as well as the bacteria *Anaplasma marginale* (18.9%), *Anaplasma* sp. (2.2%), *Ehrlichia* sp. (6.6%), *Ehrlichia minasensis* (0.4%), and *Aureimonas altamirensis* (1.3%). *Anaplasma marginale* was the most prevalent. Codetections were common, with multiple ticks harboring two or more pathogens. Some double detection occurred more frequently than expected by chance.

**Conclusions:**

This study presents the first comprehensive evidence of diverse tick-borne pathogens circulating in cattle ticks from Lao PDR, revealing high genetic similarity to globally recognized strains alongside distinct region-specific detection patterns. Notably, it also constitutes the first report of *A. marginale*, *E. minasensis*, *B. bigemina*, *B. bovis*, and *Theileria* sp. in cattle ticks in the country. These findings underscore the urgent need for integrated tick and pathogen surveillance within a One Health framework, with significant implications for regional disease monitoring, livestock health management, and zoonotic risk mitigation.

**Graphical Abstract:**

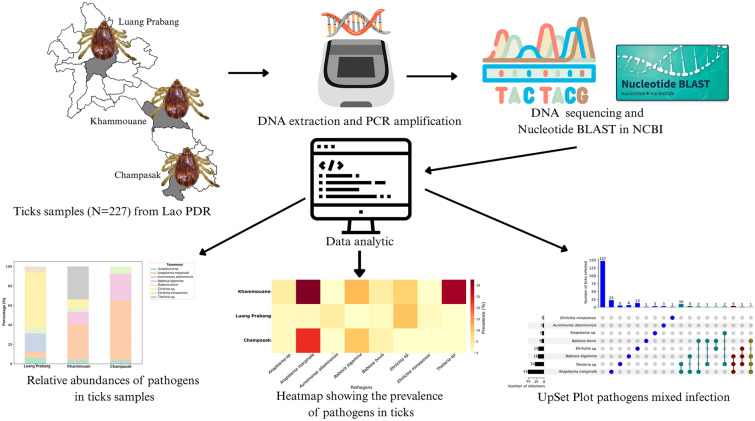

**Supplementary Information:**

The online version contains supplementary material available at 10.1186/s13071-025-07167-2.

## Background

Ticks are hematophagous ectoparasites that play a pivotal role in the transmission of a wide array of pathogens affecting both animals and humans [1]. In livestock, tick-borne diseases (TBDs) pose a major constraint to production, especially in tropical and subtropical regions where warm climates and extensive farming practices promote year-round tick survival and transmission cycles. In Southeast Asia, particularly in the Lao People’s Democratic Republic (Lao PDR), a country marked by the winding Mekong River and rugged mountainous terrain, the cattle industry plays a vital role in sustaining rural livelihoods and ensuring food security. However, research and surveillance on TBDs in the country remain limited. Cattle ticks, notably *R. microplus*, serve as vectors for several hemoparasitic and bacterial agents of veterinary and potentially zoonotic importance. Pathogens such as *A. marginale*, *B. bigemina*, *B. bovis*, and *Theileria* spp. are well-established causes of bovine anaplasmosis, babesiosis, and theileriosis, respectively, diseases that lead to fever, hemolytic anemia, decreased milk yield, reproductive losses, and high mortality, especially in naive or untreated animals [2–5].

In addition to their economic impact, several tick-borne pathogens pose an increasing public health concern owing to their zoonotic potential. *Anaplasma phagocytophilum* and *Ehrlichia chaffeensis* are known to infect humans, causing granulocytic anaplasmosis and monocytic ehrlichiosis, respectively [4–6]. Although *A. marginale* is traditionally regarded as nonzoonotic, molecular evidence suggests that various *Anaplasma* species, including as-yet unidentified strains, may have a broader host range than previously recognized. Similarly, *E. minasensis*, a species recently isolated from bovine and tick samples in South America and Asia, has pathogenic potential in both veterinary and human contexts [7, 8]. Of additional concern is *Au. altamirensis*, an environmental bacterium first identified in cave microbiota, which has subsequently been detected in hospital isolates [9, 10]. While its pathogenic status remains unclear, its presence in ticks suggests the need for further investigation, especially in undersurveyed regions including Lao PDR.

Despite these concerns, there is a critical lack of data regarding the diversity, prevalence, and co-infection patterns of tick-borne pathogens in Lao PDR. The geographic variability, seasonal dynamics, and ecological heterogeneity of Lao PDR suggest that distribution of tick-borne pathogens may vary considerably across regions. Coinfections, the presence of multiple pathogens within single tick vectors, further complicate diagnosis, disease management, and control strategies, as well as potentially altering disease dynamics and vector competence.

To address these gaps, the present study aimed to conduct a comprehensive molecular survey of cattle ticks collected from three major ecological zones in Lao PDR: the northern, central, and southern regions. Using polymerase chain reaction (PCR)-based molecular diagnostics, we targeted a panel of high-priority pathogens including *Ehrlichia* sp., *Anaplasma* sp., *B. bigemina*, *B. bovis*, and *Theileria* sp. This study aimed to determine the prevalence and diversity of pathogens in cattle ticks, assess patterns of co-infection, and evaluate spatial variation in pathogen distribution across regions. The findings are expected to provide essential baseline data for epidemiological modelling, risk assessment, and the development of integrated tick-borne disease control strategies in Lao PDR and the broader Mekong region.

## Methods

### Tick collection and morphological identification of tick species

**Fig. 1:**
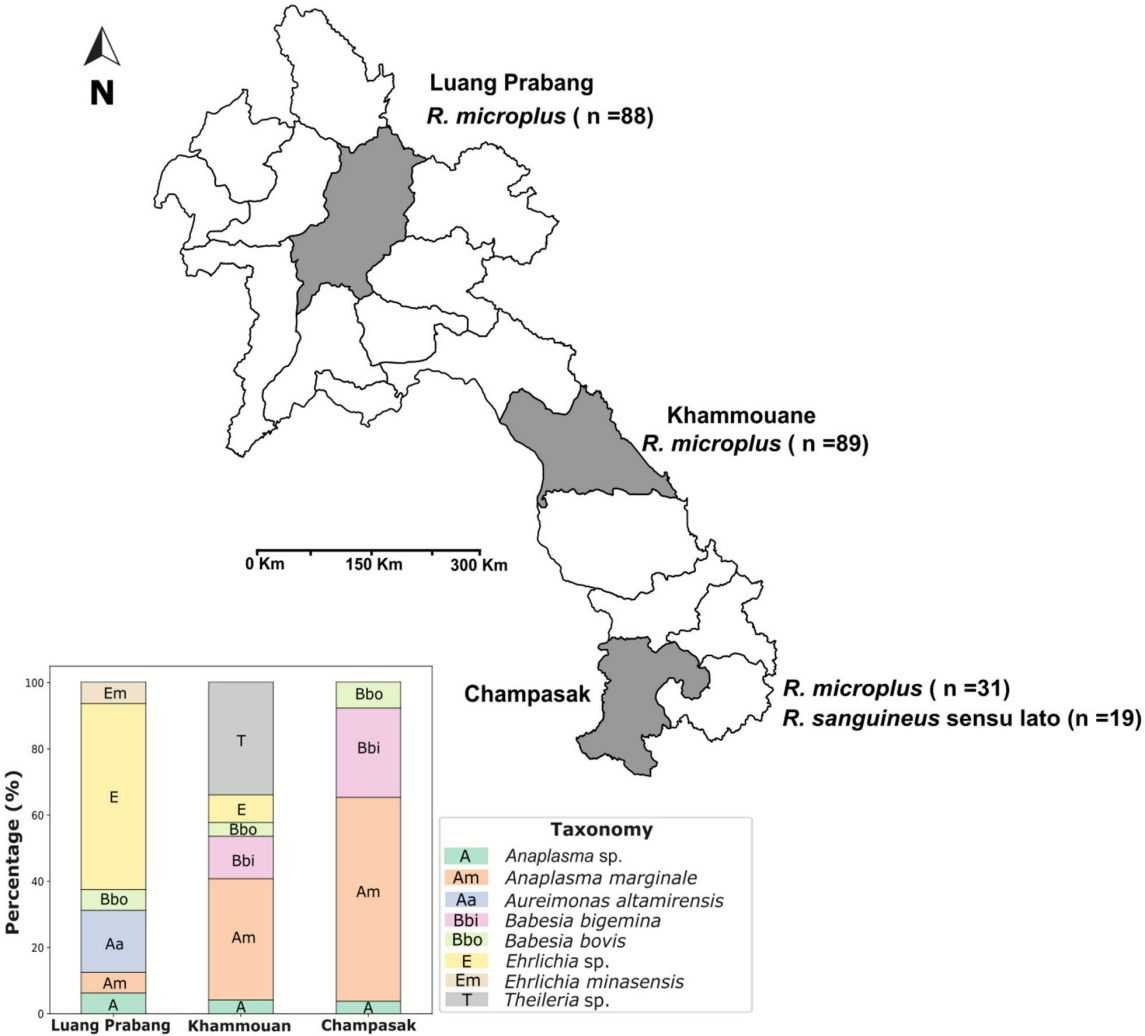
Sampling locations and pathogen infection profiles of ticks collected from cattle in the Lao People’s Democratic Republic. The map shows the three provinces where tick specimens were collected: Luang Prabang (north), *Rhipicephalus microplus* (*n* = 88); Khammouane (central), *R. microplus* (*n* = 89); and Champasak (south), *R. microplus* (*n* = 31) and *Rhipicephalus sanguineus* sensu lato (*n* = 19). The accompanying stacked bar plot represents the proportion of infected ticks hosting each pathogen in each province, highlighting regional variations in pathogen proportions

Ticks were collected from a total of 63 cattle across three provinces in Lao PDR: Luang Prabang (northern region), Khammouane (central region), and Champasak (southern region). The number of cattle sampled and the tick species identified from each region are presented in Table 1. A total of 227 ticks were collected, predominantly *R. microplus* (*n* = 208), which was found in all three provinces. *Rhipicephalus sanguineus* sensu lato (*n* = 19) was detected exclusively in Champasak (Fig. 1). Ticks were manually removed from the host’s skin using sterile forceps and preserved in 90% ethanol. Morphological identification was performed under a stereomicroscope using standard taxonomic keys [11–13].

**Table 1:** Number of tick species collected from cattle in three provinces of Lao PDR

Province/region	Number of cattle (*n*)	Tick species	
		*Rhipicephalus microplus* (*n*)	*Rhipicephalus sanguineus* sensu lato (*n*)
Luang Prabang (northern)	39	88	–
Khammouane (central)	18	89	–
Champasak (southern)	6	31	19
Total	63	208	19

### DNA extraction

Following morphological identification, individual ticks were washed with deionized water and subjected to genomic DNA extraction using the NucleoSpin^®^ Tissue kit (Macherey–Nagel GmbH & Co. KG, Germany), according to the manufacturer’s instructions. DNA concentrations were measured using a NanoDrop™ One spectrophotometer (Thermo Fisher Scientific, Waltham, MA), and DNA samples were stored at −20 °C until further use.

### PCR amplification and DNA sequencing

#### Molecular confirmation of tick species

After morphological identification, a total of seven ticks were selected for molecular confirmation on the basis of species and geographic representation. Specifically, two *R. microplus* specimens were chosen from each of the three provinces (Luang Prabang, Khammouane, and Champasak), and one *R. sanguineus* s. l. specimen was selected from Champasak, where this species was exclusively found.

A portion of the cytochrome c oxidase subunit I (*cox*1) gene of these ticks was amplified using the primers S0725 (F1: 5′-TAC TCT ACT AAT CAT AAA GAC ATT GG-3′) and S0726 (R1: 5′-CCT CCT CCT GAA GGG TCA AAA AAT GA-3′), which generates a ~656 bp product [14]. PCR reactions (25 μL) contained 12.5 μL of 2 × GoTaq^®^ Green Master Mix (Promega, Madison, WI), 0.5 μL of each primer (10 pmol), 2 μL of template DNA, and 9.5 μL of nuclease-free water. Cycling conditions were: initial denaturation at 94 °C for 4 min; 35 cycles of 94 °C for 30 s, 50 °C for 30 s, and 72 °C for 45 s; followed by a final extension at 72 °C for 7 min. PCR products were visualized on 1.5% agarose gels and positive amplicons were submitted for sequencing to BIONICS Co., Ltd. (Seoul, South Korea).

#### Detection of bacterial and protozoan pathogens in tick samples

The presence of *Anaplasma* sp., *Aureimonas* sp., *Babesia* sp., *Ehrlichia* sp., and *Theileria* sp. was assessed in DNA from all 227 tick samples using conventional PCR and sequence analyses. The total PCR reaction volume was 25 μL, containing 12.5 μL of 2 × GoTaq^®^ Green Master Mix (Promega), 0.5 μL of each primer (10 pmol) (pathogen-specific primers presented in Table 2), 2 μL DNA template, and 9.5 μL nuclease-free water. Thermal cycling consisted of an initial denaturation at 94 °C for 4 min, followed by 35 cycles of 94 °C for 30 s, annealing at temperatures presented in Table 2 for 30 s, and 72 °C for 30 s, with a final extension at 72 °C for 7 min. Amplicons were separated on 1% agarose gels stained with RedSafe^™^ Nucleic Acid Staining Solution (iNtRON Biotechnology, Korea), and positive bands were sequenced by BIONICS Co., Ltd in both directions using the PCR primers as sequencing primers.

**Table 2:** Primers used for tick-borne pathogen detection in this study

Pathogen	Target gene	Primer name	Sequence (5′−3′)	Product size (bp)	Annealing temperature (°C)	Reference
*Ehrlichia* spp./*Anaplasma* spp.	16S rRNA	EHR1F	GAACGAACGCTG GCGGCAAGC	686	60	[43]
		EHR2R	AGTAYCGRACCAGAT AGCCGC			
*Babesia bigemina*	Rhoptry-associated protein 1a	Bbi-RAP1a-879F	GAGTCTGCCAAATCCTTAC	879	50	[44]
		Bbi-RAP1a-879R	TCCTCTACAGCTGCTTCG			
*Babesia bovis*^a^	Spherical body protein 2	BboSBP2-1236F	CTGGAAGTGGATCTCATGCAACC	1236	52	[45]
		BboSBP2-1236R	TCACGAGCACTCTACGGCTTTGCAG			
		BboSBP2-580F	GAATCTAGGCATATAAGGCAT	580	45	
		BboSBP2-580R	ATCCCCTCCTAAGGTTGGCTAC			
*Theileria* spp.	18S rRNA	Piro1-S	CTTGACGGTAGGGTATTGGC	1400	48	[46]
		Piro3-AS	CCTTCCTTTAAGTGATAAGGTTCAC			

^a^ Nested PCR was employed for the detection of *Babesia bovis*

rRNA, ribosomal RNA

### DNA sequence analysis

#### Tick analysis

Forward and reverse nucleotide sequences were assembled using BioEdit [15]. The consensus contigs were then aligned and analyzed using the BLASTn tool against the National Center for Biotechnology Information (NCBI) nucleotide database searching for highly similar sequences. Final contigs from this study were subsequently submitted to GenBank through the submission tool provided by the NCBI.

#### Pathogens in tick analysis

The sequences were aligned with those of related pathogens retrieved from the NCBI database using the ClustalW algorithm implemented in MEGA version 11 with default parameter settings. Phylogenetic analysis was then conducted using the maximum likelihood method on the basis of the best-fit model of each pathogen in MEGA version 11 [16]. The Kimura 2-parameter model with a gamma distribution (K2 + G) [17] was applied for *Anaplasma* sp., *Ehrlichia* sp., *B. bigemina*, *B. bovis*, and *Theileria* sp., while the Hasegawa–Kishino–Yano model with a gamma distribution (HKY + G) [18] was used for *Aureimonas* sp. Bootstrap support values were estimated from 1000 replicates.

### Data analysis

To assess differences in pathogen infection profiles among ticks from each province (Luang Prabang, Khammouane, and Champasak), a binary matrix (1 = infected, 0 = not infected) for eight pathogens across 227 samples was initially prepared. Because the number of samples per province was unbalanced, subsampling was applied to equalize the sample size per province, resulting in a balanced dataset. Permutational analysis of multivariate dispersions (PERMDISP) was assessed using Bray–Curtis dissimilarities to examine differences in community dispersion among provinces. The analysis was implemented in Python (v3.11.7) (f_oneway, pdist and squareform from SciPy v1.11.4) [19]. PERMANOVA was then performed on the balanced dataset using Bray–Curtis dissimilarity with 999 permutations [20] (permanova function from scikit-bio v0.6.3 [21]).

A stacked bar plot was generated to visualize the composition of pathogen taxa across the three geographic groups. Data were organized with taxa as rows and sampling locations as columns. Relative abundances were calculated as percentages on the basis of the total counts within each group. The plot was constructed using matplotlib (v3.8.0) [22], with pastel colors applied from the seaborn palette to distinguish taxa. The *x*-axis represents the geographic groups, while the *y*-axis shows the proportion of infected ticks hosting each taxon in each geographic region.

To visualize spatial differences in infection prevalence, a heatmap was generated to display the percentage of infected samples for each pathogen across the three regions. Prevalence was calculated as the proportion of positive samples relative to the total number of samples per region. The heatmap was constructed using seaborn (v0.12.2) [23], with color gradients representing infection prevalence levels.

For the analysis of double infections, the chi-squared test of independence was conducted using the chi2_contingency function from the scipy.stats library (v1.11.4) [19] in Python (v3.11.7). The chi-squared statistic, degrees of freedom, and associated *P*-value were calculated for each test. Statistical significance was defined as *P* < 0.05. Pathogen co-infection patterns were further visualized with UpSet plots on the basis of the same binary matrix, using the upset plot library (v0.6.0) [24] to illustrate frequencies and combinations of single and multiple infections across samples. All data processing utilized pandas (v2.2.3) [25], and computations were performed with standard Python scientific libraries.

## Results

### Identification of ticks

A total of 227 cattle ticks were collected from 63 cattle in Luang Prabang, Khammouane, and Champasak, Lao PDR. Tick species were morphologically identified as *R. microplus* (*n* = 208) and *R. sanguineus* s. l. (*n* = 19). *Rhipicephalus microplus* was the dominant species across all collection sites, whereas *R. sanguineus* s. l. was detected only in Champasak Province. Eight ticks, randomly selected on the basis of morphological identification, were confirmed by sequencing of the *cox*1 gene. All molecularly identified tick species were consistent with their corresponding morphological identifications. Our seven *R. microplus* sequences (GenBank accession nos. PV746283–PV746288, and PV746290) exhibited 100% identity with sequences previously reported from ticks collected in northeastern provinces of Thailand, adjacent to Lao PDR. The relevant Thai sequences available in GenBank include accession nos. OM761020 (Loei), OM761022 (Mukdahan), OM761031 (Roi-Et), OM761039, and OM761041 (Bueng Kan). Our one *Rhipicephalus linnaei* sequence (GenBank accession no. PV746289) showed 100% identity with a sequence from Belize, Central America (GenBank accession no. ON134429).

### Molecular characterization of tick-borne pathogens

Nucleotide sequence analysis of tick DNA revealed multiple bacterial and protozoan pathogens. Among the bacterial species, *A. marginale* was the most frequently detected (43 samples out of 227 samples). All sequences showed 100% identity with a sequence (JQ839012) previously reported from a tick in the Philippines. In addition, five sequences were identical (100%) with an *Anaplasma* sp. sequence obtained from an impala in South Africa (OQ909463). In total, 15 sequences matched *Ehrlichia* sp., showing 100% similarity with a sequence in *R.* (*Boophilus*) *microplus* from China (AF414399), and one sample was identified as *E. minasensis* with 99.58% identity with a Brazilian tick isolate (NR_148800). Three sequences corresponded to *Au. altamirensis*, two of which showed 100% identity and one with 99.68% similarity to a sequence previously isolated from a frog in Madagascar (MF523847). The phylogenetic tree of bacterial pathogens is provided in Additional File 1: Supplementary Fig. 1.

Among the protozoan parasites, *B. bigemina* was identified in various samples showing high sequence similarity (99.29–99.76%) to reference sequences from Syrian cattle (AB617643) and the Mexican isolate (M60878). For *B. bovis*, six sequences were detected, with percentage similarity ranging from 99.81 to 100% compared with sequences from Thailand (KU764507 and AB772317), Philippines (JX648555), Benin (KX685393), and Vietnam (AB742544). Supplementary Fig. 2 displays the phylogenetic tree of *Babesia* species.

In addition, 24 nucleotide sequences showed 100% identity with a *Theileria* sp. sequence from cattle in Thailand (AB000270). The phylogenetic relationships of *Theileria* species are shown in Supplementary Fig. 3. These findings confirm the presence of multiple tick-borne pathogens with high genetic similarity to known strains from various geographic regions (Table 3).

**Table 3:** Matches of sequences from pathogens found in cattle ticks from Lao People's Democratic Republic with existing sequences in the GenBank database

Pathogens	Gene	Accession number (this study)	No. of ticks positive	Reference accession numbers (GenBank)	% similarity	Host/country
***Anaplasma*** **sp.**	16s rRNA	PV746476	5	OQ909463	100	Impala/South Africa
*A. marginale*	16s rRNA	PV746475	43	JQ839012	100	Tick/Philippines
***Ehrlichia*** **sp.**	16s rRNA	PV746477	15	AF414399	100	Tick/China
*E. minasensis*	16s rRNA	PV746478	1	NR_148800	99.58	Tick/Brazil
*Aureimonas altamirensis*	16s rRNA	PV746485	2	MF523847	100	Frog/Madagascar
		PV746486	1	MF523847	99.68	Frog/Madagascar
*Babesia bigemina*	*Rhoptry-associated protein 1a*	PV751025	3	AB617643	99.76	Cattle/Syria
		PV751026	3	AB617643	99.52	Cattle/Syria
		PV751027	2	AB617643	99.29	Cattle/Syria
		PV751029	7	AB617643	99.41	Cattle/Syria
		PV751028	1	M60878	99.41	Cattle/Mexico
*B. bovis*	*Spherical body protein 2*	PV751020	2	KU764507	100	Cattle/Thailand
		PV751021	1	JX648555	100	Cattle/Philippines
		PV751022	1	KX685393	100	Cattle/Benin
		PV978366	1	AB742544	100	Cattle/Viet Nam
		PV751024	1	AB772317	99.81	Cattle/Thailand
***Theileria*** **sp.**	18s rRNA	PV746293	24	AB000270	100	Cattle/Thailand

### Geographical distribution of pathogens in cattle ticks

**Fig. 2:**
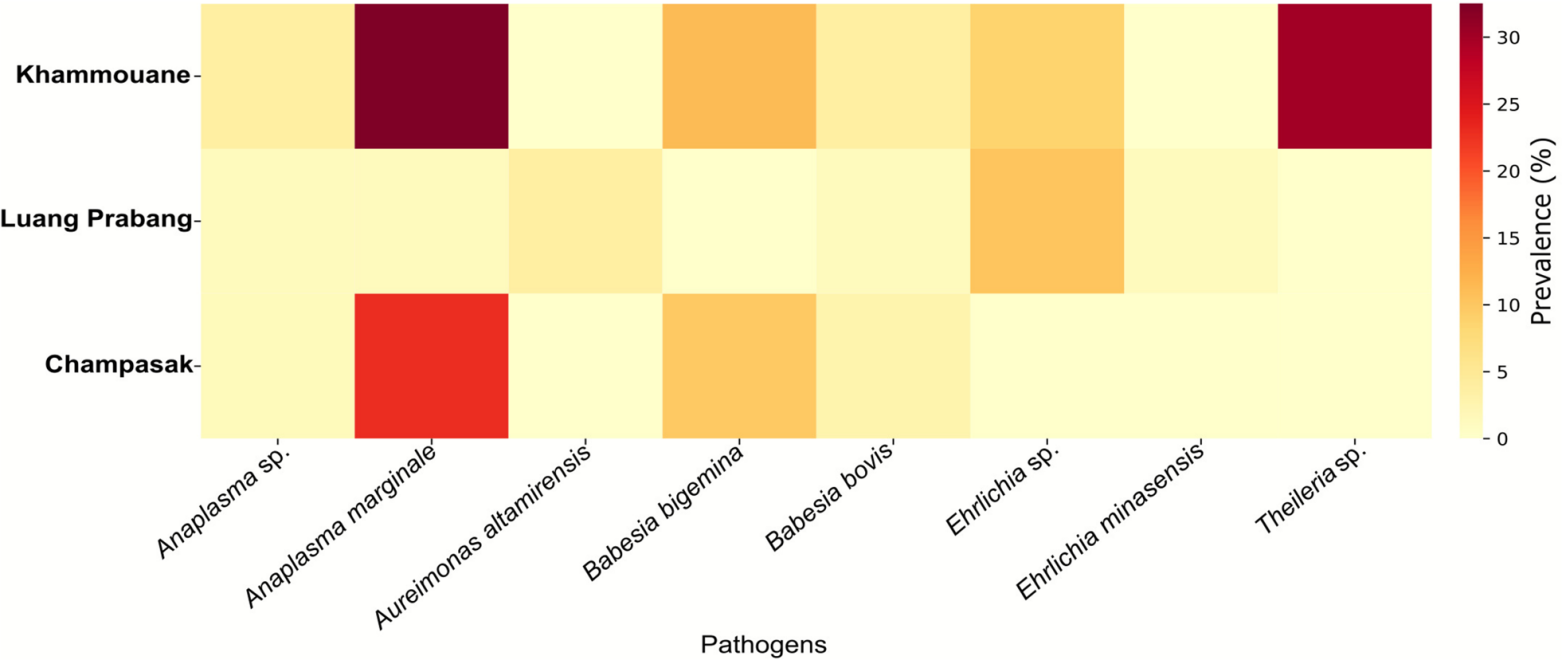
Heat map showing the prevalence of pathogens in cattle tick vectors in three different provinces of Lao PDR: Luang Prabang (*n* = 88), Khammouane (*n* = 89), and Champasak (*n* = 50). The color intensity represents the number of infected ticks, with darker shades indicating higher infection levels. Pathogen names are listed on the *x*-axis and provinces on the *y*-axis

From the initial 227 tick samples, the number of samples per province was unbalanced, with 88 from Luang Prabang, 89 from Khammouane, and 50 from Champasak. To account for this imbalance and reduce potential bias in PERMANOVA due to unequal dispersion (PERMDISP *F* = 7.282, *P* = 0.0009), a subsampling approach was applied to equalize the number of samples per province (*n* = 50 each), resulting in a balanced dataset of 150 samples. PERMANOVA analysis on the balanced dataset revealed statistically significant differences in the overall pathogen infection profiles among the geographic areas (pseudo-*F* = 4.495, *P* = 0.001). These results suggest that pathogen composition varied by location, indicating spatial structuring in tick populations. The heat map visualization (Fig. 2) demonstrates distinct differences in infection patterns among provinces. The highest prevalence of *A. marginale* was observed in Khammouane and Champasak, while *Theileria* sp. was detected exclusively in Khammouane.

**Fig. 3:**
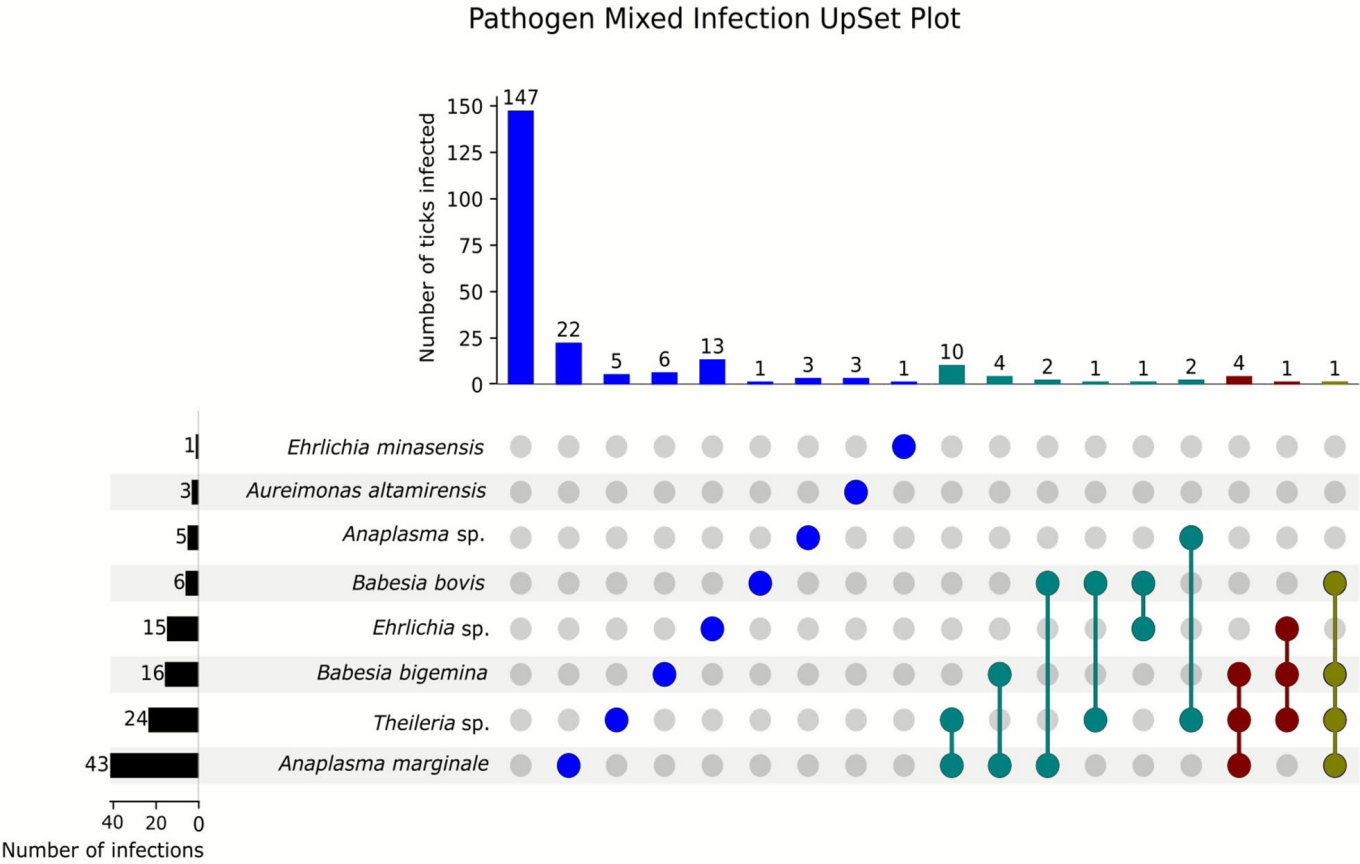
UpSet plot showing codetection patterns of pathogens among 227 tick specimens. Vertical bars represent the number of samples with each unique pathogen combination; connected nodes indicate specific codetections. Horizontal bars show total infections per pathogen. The most frequent codetection was between *Anaplasma marginale* and *Theileria* spp., followed by combinations involving *Babesia bigemina*, *Babesia bovis*, and *Ehrlichia* spp. Detection categories are color-coded as single (blue), double (green), triple (purple), and quadruple (yellow) detections

The most frequently detected pathogen was *A. marginale*, identified in 43 (18.9%) of ticks, followed by *Theileria* sp. (24; 10.6%), *B. bigemina* (16; 7%), *Ehrlichia* sp. (15; 6.6%), *B. bovis* (6; 2.6%), *Anaplasma* sp. (5; 2.2%), *Au. altamirensis* (3; 1.3%), and *E. minasensis* (1; 0.4%). Single detection was the most frequently observed in ticks, with an overall prevalence of 23.6%. Among these, *A. marginale* was the most common pathogen (22; 9.7%), followed by *Ehrlichia* sp. (13; 5.7%), *B. bigemina* (6; 2.6%), and *Theileria* sp. (5; 2.2%). Lower prevalences were recorded for *Au. altamirensis* (3; 1.3%), *Anaplasma* sp. (3; 1.3%), *B. bovis* (1; 0.4%), and *E. minasensis* (1; 0.4%) (Additional File 2: Supplementary Table 1). Mixed infections were further examined using an UpSet plot, shown in Fig. 3. Double detections were observed in several tick specimens. The most common combination was *A. marginale* and *Theileria* sp. (10; 4.4%), followed by *A. marginale* and *B. bigemina* (4; 1.8%), *A. marginale* and *B. bovis* (2; 0.9%), *Anaplasma* sp. and *Theileria* sp. (2; 0.9%), *B. bovis* and *Theileria* sp. (1; 0.4%), and *B. bovis* and *Ehrlichia* sp. (1; 0.4%) (Additional File 2: Supplementary Table 2 and Fig. 3). A chi-squared test indicated a significantly higher frequency than expected by chance of co-infections by *A. marginale* and *Theileria* sp. (*χ*^2^ = 30.06, *df* = 1, *P* < 0.001), and by *A. marginale* and *B. bigemina* (*χ*^2^ = 13.10, *df* = 1, *P* = 0.0003). In contrast, no statistically significant associations were detected between *A. marginale* and *B. bovis* (*χ*^2^ = 2.07, *df* = 1, *P* = 0.150), *Anaplasma* sp. and *Theileria* sp. (*χ*^2^ = 2.04, *df* = 1, *P* = 0.153), *B. bovis* and *Ehrlichia* sp. (*χ*^2^ = 0.03, *df* = 1, *P* = 0.863), or *B. bovis* and *Theileria* sp. (*χ*^2^ = 1.36, *df* = 1, *P* = 0.244). However, small sample size and corresponding low statistical power could have influenced these results.

Multiple detections involving three or more pathogens were also recorded. These included *A. marginale*, *B. bigemina*, and *Theileria* sp. (4; 1.8%); *Ehrlichia* sp., *B. bigemina*, and *Theileria* sp. (1; 0.4%); and a quadruple detection involving *A. marginale*, *B. bigemina*, *B. bovis*, and *Theileria* sp. (1; 0.4%) (Additional File 2: Supplementary Table 2 and Fig. 2).

## Discussion

This study provides the first comprehensive molecular characterization of pathogens in cattle ticks from across the northern, central, and southern regions in Lao PDR. The integration of morphological and molecular methods confirmed the identification of *R. microplus* and *R. linnaei*, consistent with regional tick fauna previously reported in Asia [1, 26, 27]. Molecular sequencing revealed a high degree of concordance between field-collected tick sequences and strains found in other countries. Sequences of *R. microplus* obtained in this study demonstrated ≥ 99–100% similarity to sequences previously reported from ticks collected in northeastern Thailand (bordering Lao PDR) and other *R. microplus* deposited in GenBank, supporting the low genetic diversity of this species across its broad distribution in Asia [11, 28, 29]. Similarly, the *R. linnaei* sequence exhibited 100% identity (ON134429) with a sequence previously reported from Belize, confirming its close genetic relationship to *R. linnaei* populations from other tropical regions. These findings are consistent with recent taxonomic updates recognizing *R. linnaei* as a distinct species within the *R. sanguineus* complex [30]. The detection of *R. linnaei* in Lao PDR broadens its known range in Southeast Asia and emphasizes the importance of molecular approaches for accurate tick taxonomy.

We report here the first molecular detection of tick-borne pathogens in *Rhipicephalus* species from Lao PDR, including *A. marginale* (18.9%), *B. bigemina* (7%), *B. bovis* (2.6%), *Ehrlichia minasensis* (0.4%), and *Au. altamirensis* (1.3%). Among these, *A. marginale* was the most prevalent bacterial pathogen detected. Screening revealed multiple bacterial and protozoan pathogens, underscoring the diverse tick-borne pathogen landscape in the region.

The *Anaplasma* sequences, primarily identified as *A. marginale*, a well-known causative agent of bovine anaplasmosis [31] were detected in *R. microplus* and *R. sanguineus* s. l. These sequences showed high similarity (> 99%) to strains from the Philippines, Taiwan (OL660541), China (HM439433), and Japan (FJ226454), suggesting low genetic diversity across Asia–Pacific [32]. In Lao PDR, *A. marginale* has not yet been reported in ticks collected from cattle; however, it has been detected in the spleen of a muntjac (*Muntiacus muntjak*) [33].

Additional *Anaplasma* sp. sequences, detected exclusively in *R. microplus* in this study, matched isolates from South African wildlife, including impalas, indicating a potentially broader geographic distribution and host range within mammals [34]. This highlights the importance of ongoing surveillance to monitor cross-species transmission risks. In Lao PDR, *Anaplasma* sp. have also been reported in *Amblyomma testudinarium*, *A. javanense*, and *Haemaphysalis* G1 ticks collected from Khammouane Province [35, 36]; however, it remains unclear whether these *Anaplasma* sp. are pathogenic to cattle.

The detection of *Ehrlichia* sp. and *E. minasensis* was restricted to *R. microplus*. The *Ehrlichia* sp. sequences were identical (100%) to *Ehrlichia* sp. (AF414399) previously reported in *R. microplus* from China, whereas *E. minasensis* sequences showed 99.58% similarity to *E. minasensis* (NR_148800) detected in *R. microplus* from Brazil, reflecting the pathogen’s expanding range and possible vector associations beyond historically described hosts [7, 8]. *Ehrlichia* sp. has been detected in *Amblyomma testudinarium*, *A. javanense*, *Haemaphysalis* G1, and *H. aborensis* ticks collected from Khammouane Province, Lao PDR [35, 36]; however, it remains unclear whether this *Ehrlichia* sp. is pathogenic to cattle. *Ehrlichia minasensis* has been reported in cattle ticks (*R. microplus*) [7] and is also known to be pathogenic to cattle [37]. Furthermore, in Lao PDR, *E. minasensis* has not previously been detected in ticks collected from cattle; thus, this study represents the first report of *E. minasensis* from cattle-associated ticks in the country.

*Aureimonas altamirensis*, though infrequently reported in tick vectors, was detected in three cases, showing high similarity with isolates from environmental and clinical sources. This finding raises questions about its ecological role and whether it represents a commensal or opportunistic pathogen. However, as the ticks were engorged, the detected pathogen DNA may originate from the host’s bloodmeal rather than true tick infection, so vector competence cannot be confirmed from these results [9, 10].

Among apicomplexan protozoans, *B. bigemina* was the most frequently detected, with sequences exhibiting high similarity to strains from Syrian and Mexican cattle, indicating that this pathogen is a widely distributed hemoparasite in tropical and subtropical climates [3]. *Babesia bovis* sequences shared similarity with isolates from Thailand, the Philippines, Benin, and Vietnam, supporting the cosmopolitan distribution of this species in cattle populations. Both *B. bovis* and *B. bigemina* are recognized as the species most commonly responsible for causing bovine babesiosis [2, 38]. Notably, the detection of *B. bigemina* and *B. bovis* in cattle ticks represents the first such report in Lao PDR.

Importantly, *Theileria* sp. sequences exhibited high identity with reference strains from Thailand, and phylogenetic analysis (shown in Additional File 1: Supplementary Fig. 3) strongly suggests that the species is *T. sinensis*. Preliminary reports indicate that *T. sinensis* may cause anemia in cattle, as a study in Malaysia found that infected animals exhibited normocytic normochromic anemia [5]. In addition, reports from cattle in Thailand (bullfighting cattle) indicated that animals infected with *T. sinensis* exhibited a significant decrease in red blood cell (RBC) counts [39]. In Lao PDR, this study represents the first report to detection of *Theileria* sp. in cattle tick vector. Its detection in Lao PDR suggests regional connectivity in tick-borne protozoan diversity and underscores the need for targeted epidemiological studies to assess its impact on livestock health in this area.

Collectively, these findings demonstrate that tick-borne pathogens in Lao PDR have high genetic similarity to strains circulating in other endemic regions, implying common sources, shared vectors, and/or historic movement of infected livestock. Multivariate statistical analysis further revealed spatial structuring in pathogen prevalence. PERMANOVA results indicated significant differences in detection profiles among ticks from northern, central, and southern regions of Lao PDR, supporting previous assertions that environmental and ecological heterogeneity influence vector–pathogen dynamics [40]. *Anaplasma marginale* remained the most prevalent single-agent detection, followed by *Theileria* sp., *B. bigemina*, and *Ehrlichia* spp. However, mixed detection, particularly those involving *A. marginale* and *Theileria* sp., were also common and have been shown to exacerbate disease severity and complicate treatment protocols [41]. Some double detection occurred more frequently than expected by chance, a finding that should prompt further research. Triple and quadruple codetections were also detected, underscoring the complex pathogen ecology within Lao PDR tick populations. Such polymicrobial infections may indicate overlapping transmission cycles or immunological tolerance within both hosts and vectors, potentially exacerbating disease emergence and clinical outcomes in livestock. These infections can reduce animal productivity and economic returns, while also posing cross-border risks through regional trade, animal movement, and shared grazing ecosystems, particularly along the borders with Thailand and Vietnam. A One Health approach integrating veterinary surveillance and transboundary cooperation is therefore essential to moderate their impact on livestock health and regional disease transmission [42].

## Conclusions

This study highlights the high prevalence and genetic diversity of tick-borne pathogens in Lao PDR and provides molecular evidence for the presence of multiple zoonotic and veterinary pathogens. The close genetic relationships with international strains underscore the potential for cross-border transmission and reinforce the need for regional surveillance frameworks. These findings contribute valuable baseline data for designing integrated vector management strategies and support One Health approaches to mitigate the impact of TBDs in Southeast Asia.

## Supplementary Information

**Supplementary Material 1: **Fig S1. Phylogenetic relationships among *Anaplasma, Ehrlichia*, and *Aureimonas* sequences obtained from cattle ticks and elsewhere, inferred using maximum likelihood analysis of 16S rRNA gene sequences. For panel, the tree was constructed using the Kimura 2-parameter model with a gamma distribution, while for panel, the Hasegawa-Kishino-Yano model with a gamma distributionwas applied. Bootstrap valuesare shown at the nodes. All DNA sequences are labeled with GenBank accession numbers, species names, country codes following the ISO 3166–1 alpha-3 standard and host. Sequences obtained in this study are shown in bold, withindicating the number of tick samples sharing identical nucleotide sequences.

**Supplementary Material 2: ** Fig S2. Phylogenetic placement of *Babesia bigemina* and *B. bovis* sequences from cattle tick samples and elsewhere, inferred using maximum likelihood analysis based on the rhoptry-associated protein 1a gene for *B. bigemina* and the spherical body protein 2 gene for *B. bovis*. Bootstrap valuesare shown at the nodes, based on the Kimura 2-parameter model with a gamma distribution. All DNA sequences are labeled with GenBank accession numbers, species names, country codes following the ISO 3166–1 alpha-3 standard and host. Sequences obtained in this study are shown in bold, withindicating the number of tick samples sharing identical nucleotide sequences.

**Supplementary Material 3: **Fig S3. Inferred phylogeny of *Theileria* spp. on the basis of 18S rRNA gene sequences obtained from cattle ticks and elsewhere. Bootstrap valuesare shown at the nodes. The tree was constructed using the Kimura 2-parameter model with a gamma distribution. All DNA sequences are labeled with GenBank accession numbers, species names, country codes following the ISO 3166–1 alpha-3 standard and host. Sequences obtained in this study are shown in bold, withindicating the number of tick samples sharing identical nucleotide sequences.

**Supplementary Material 4: **Table S1. Prevalence of tick-borne pathogens detected in cattle ticks collected from Lao People's Democratic Republic.

**Supplementary Material 5: **Table S2. Double and multiple infections detected in cattle ticks in Lao People's Democratic Republic.

## Data Availability

All data generated or analyzed during this study are included in this article and additional files.

## Abbreviations

*cox**1*: Cytochrome c oxidase subunit I
NCBI: National Center for Biotechnology Information
PCR: Polymerase chain reaction
PERMANOVA: Permutational multivariate analysis of variance
PERMDISP: Permutational analysis of multivariate dispersions
