# Supplementary figures and images for: Molecular detection of tick-borne pathogens in cattle ticks from the Lao People’s Democratic Republic
Source: Parasit Vectors. 2025 Dec 6;19:21. doi: 10.1186/s13071-025-07167-2 (PMC12797912; doi:10.1186/s13071-025-07167-2)

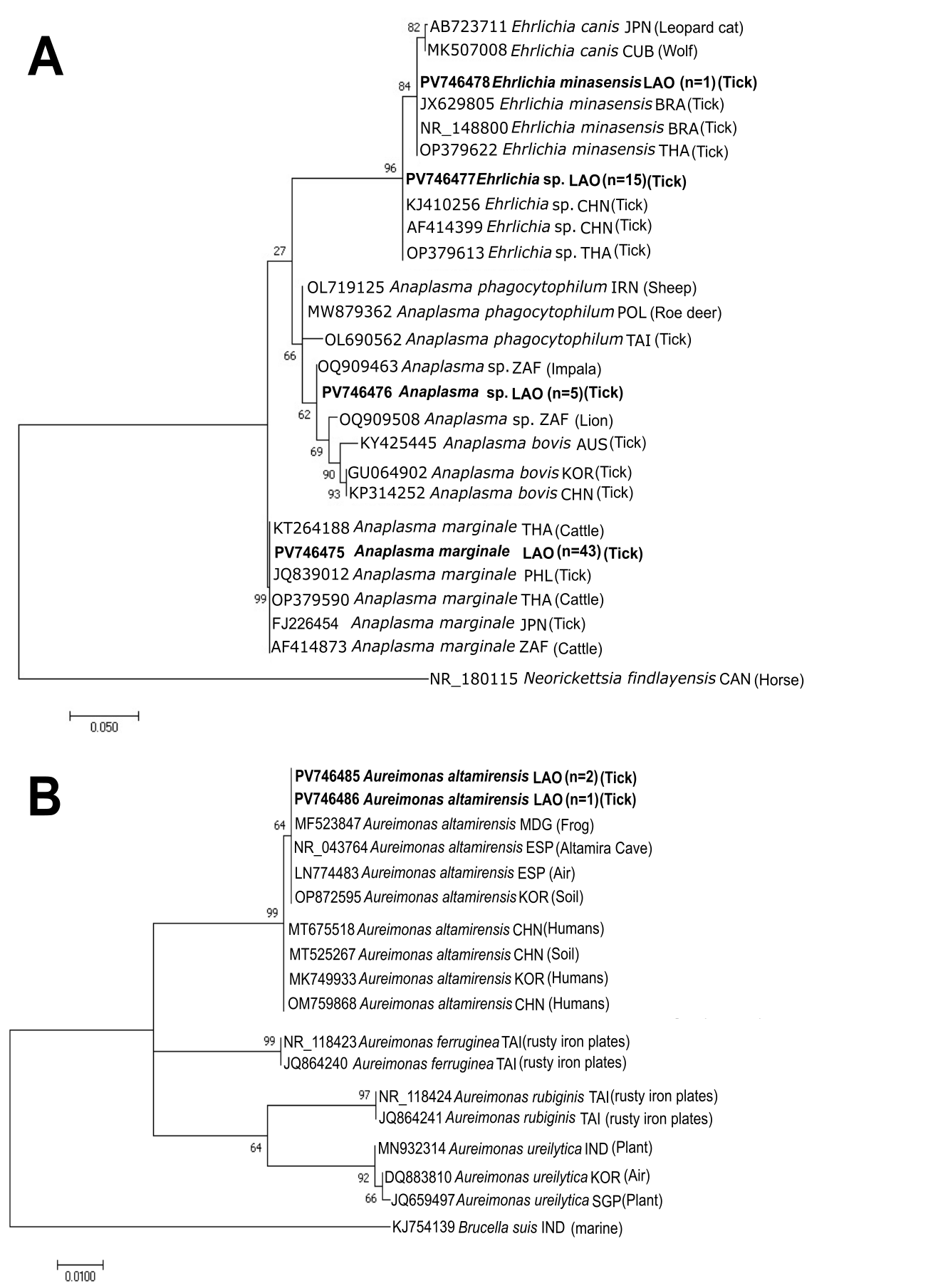

Supplement: Supplementary file 1 — Supplementary Material 1: Fig S1. Phylogenetic relationships among Anaplasma, Ehrlichia, and Aureimonas sequences obtained from cattle ticks and elsewhere, inferred using maximum likelihood analysis of 16S rRNA gene sequences. For panel, the tree was constructed using the Kimura 2-parameter model with a gamma distribution, while for panel, the Hasegawa-Kishino-Yano model with a gamma distributionwas applied. Bootstrap valuesare shown at the nodes. All DNA sequences are labeled with GenBank accession numbers, species names, country codes following the ISO 3166–1 alpha-3 standard and host. Sequences obtained in this study are shown in bold, withindicating the number of tick samples sharing identical nucleotide sequences. [file 13071_2025_7167_MOESM1_ESM.tiff]

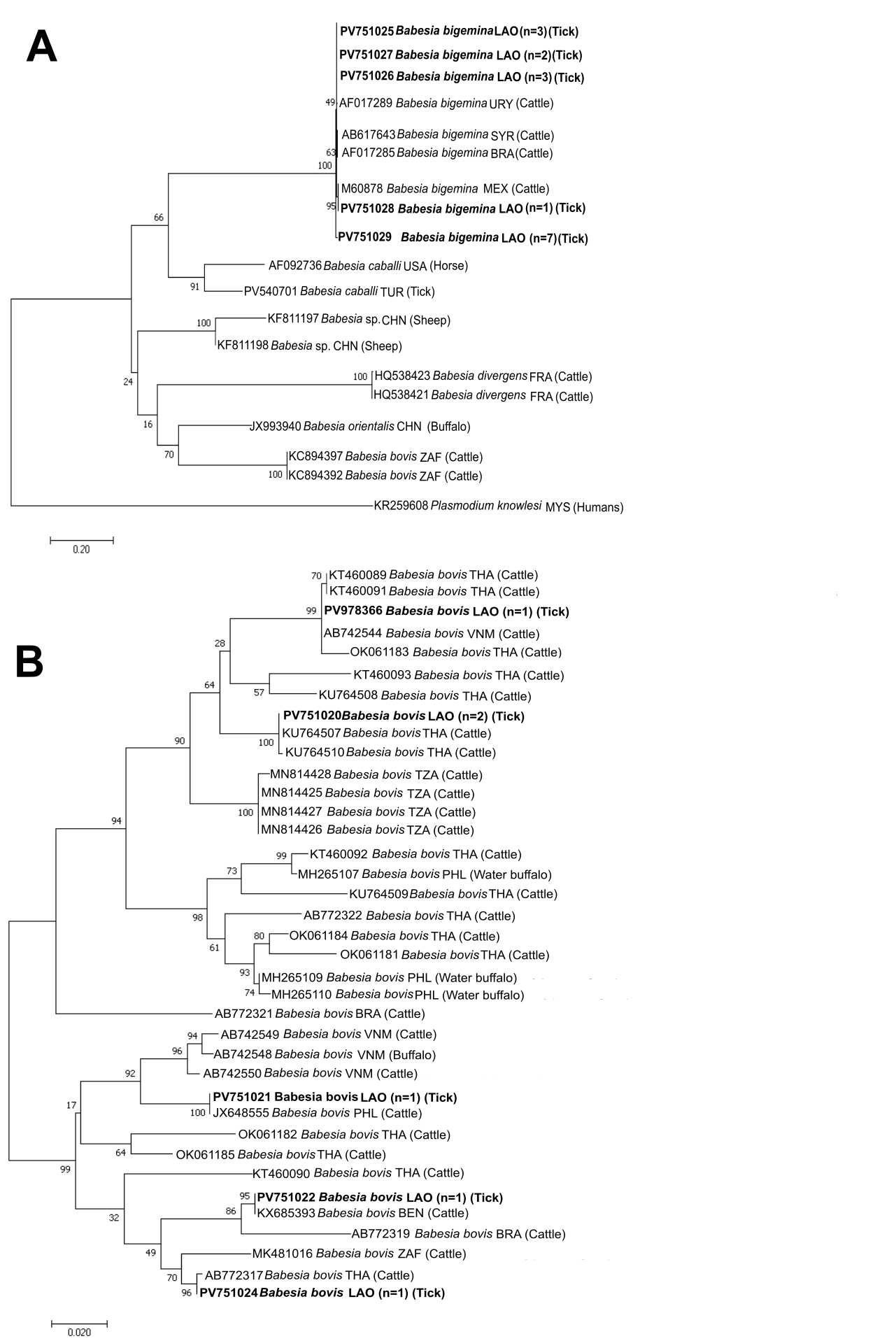

Supplement: Supplementary file 2 — Supplementary Material 2: Fig S2. Phylogenetic placement of Babesia bigemina and B. bovis sequences from cattle tick samples and elsewhere, inferred using maximum likelihood analysis based on the rhoptry-associated protein 1a gene for B. bigemina and the spherical body protein 2 gene for B. bovis. Bootstrap valuesare shown at the nodes, based on the Kimura 2-parameter model with a gamma distribution. All DNA sequences are labeled with GenBank accession numbers, species names, country codes following the ISO 3166–1 alpha-3 standard and host. Sequences obtained in this study are shown in bold, withindicating the number of tick samples sharing identical nucleotide sequences. [file 13071_2025_7167_MOESM2_ESM.tiff]

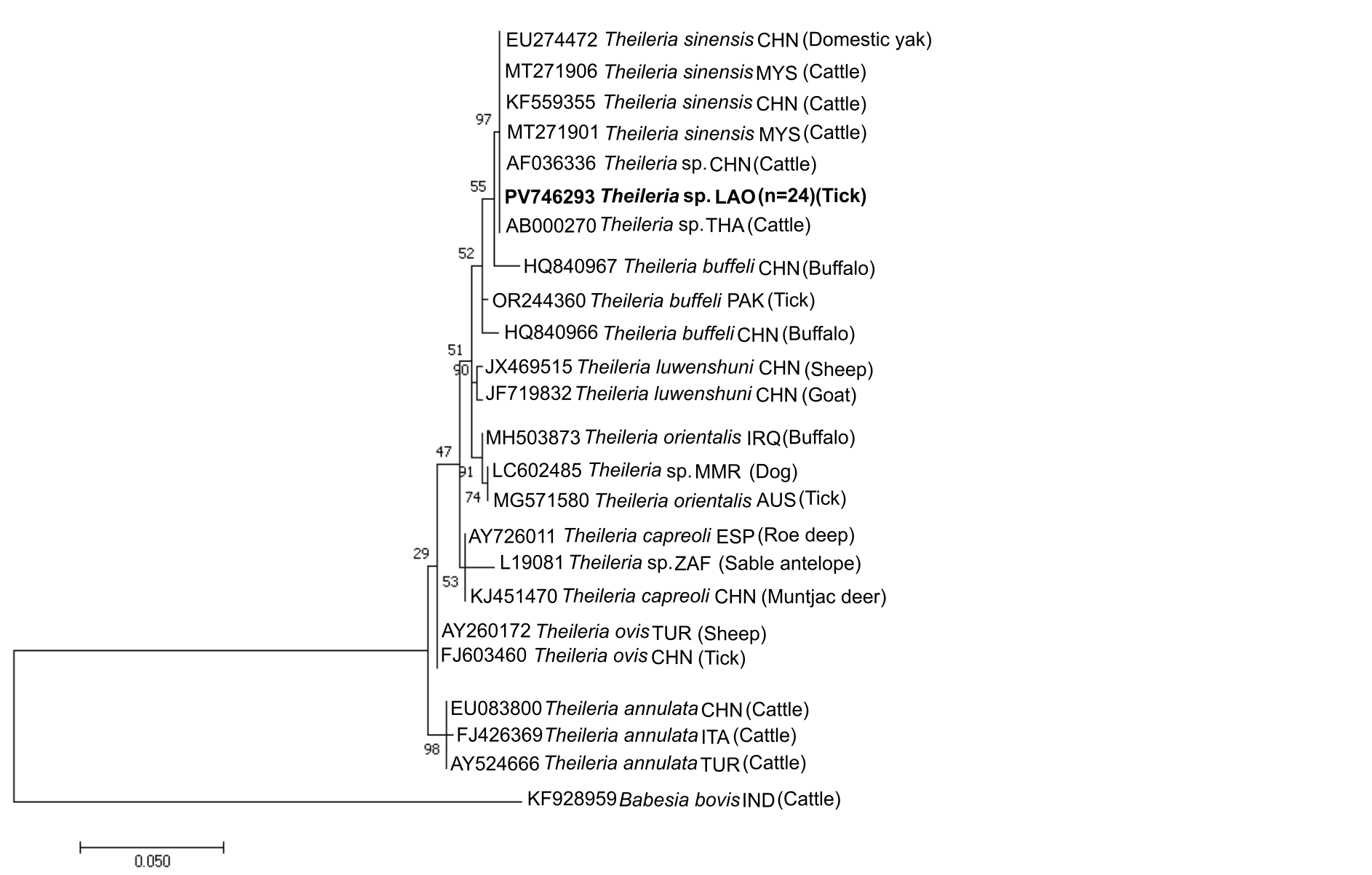

Supplement: Supplementary file 3 — Supplementary Material 3: Fig S3. Inferred phylogeny of Theileria spp. on the basis of 18S rRNA gene sequences obtained from cattle ticks and elsewhere. Bootstrap valuesare shown at the nodes. The tree was constructed using the Kimura 2-parameter model with a gamma distribution. All DNA sequences are labeled with GenBank accession numbers, species names, country codes following the ISO 3166–1 alpha-3 standard and host. Sequences obtained in this study are shown in bold, withindicating the number of tick samples sharing identical nucleotide sequences. [file 13071_2025_7167_MOESM3_ESM.tiff]
