# Supplementary material for: Molecular detection of tick-borne pathogens in cattle ticks from the Lao People’s Democratic Republic
Source: Parasit Vectors. 2025 Dec 6;19:21. doi: 10.1186/s13071-025-07167-2 (PMC12797912; doi:10.1186/s13071-025-07167-2)
Supplement: Supplementary file 4 — Supplementary Material 4: Table S1. Prevalence of tick-borne pathogens detected in cattle ticks collected from Lao People's Democratic Republic. [file 13071_2025_7167_MOESM4_ESM.docx]

Table S1. Prevalence of tick-borne pathogens detected in cattle ticks collected from Lao People's Democratic Republic

| Location | Tick  species | Number of ticks | Number of positive ticks per pathogen species (%) | | | | | | | | Number of single infections  (% of total positive prevalence) | | | | | | | |
| --- | --- | --- | --- | --- | --- | --- | --- | --- | --- | --- | --- | --- | --- | --- | --- | --- | --- | --- |
|  |  |  | A | Am | Aa | Bbi | Bbo | E | Em | T | A | Am | Aa | Bbi | Bbo | E | Em | T |
| LPB | *Rhipicephalus microplus* | 88 | 1  (1.1) | 1  (1.1) | 3  (3.4) | - | 1  (1.1) | 9  (10.2) | 1  (1.1) | - | 1 (1.1) | 1  (1.1) | 3  (3.4) | - | 1  (1.1) | 8  (9.1) | 1  (1.1) | - |
| KMN | *R. microplus* | 89 | 3  (3.4) | 26  (29.2) | - | 9  (10.1) | 3  (3.4) | 6  (6.7) | - | 24  (27) | 1  (1.1) | 9  (10.1) | - | 1  (1.1) | - | 5  (5.6) | - | 5  (5.6) |
| CPS | *R. microplus* | 31 | 1  (3.2) | 15  (48.4) | - | 7  (22.6) | 2  (6.4) | - | - | - | 1  (3.2) | 12  (38.7) | - | 5  (16.1) | - | - | - | - |
| CPS | *Rhipicephalus linnaei* | 19 | - | 1(5.3) | - | - | - | - | - | - |  |  |  |  |  |  |  |  |
| Total |  | 227 | 5  (2.2) | 43  (18.9) | 3  (1.3) | 16  (7) | 6  (2.6) | 15  (6.6) | 1  (0.4) | 24  (10.6) | 3  (1.3) | 22  (9.7) | 3  (1.3) | 6  (2.6) | 1  (0.4) | 13  (5.7) | 1  (0.4) | 5  (2.2) |

Abbreviations: LPB: Luang Prabang, KMN: Khammouane, CPS: Champasak, A: *Anaplasma* sp., Am: *Anaplasma marginale,* Aa: *Aureimonas altamirensis*, Bbi: *Babesia bigemina*, Bbo: *Babesia bovis*, E: *Ehrlichia* sp*.*, Em: *Ehrlichia minasensis*, T: *Theileria* sp.
