# Supplementary material for: Molecular detection of tick-borne pathogens in cattle ticks from the Lao People’s Democratic Republic
Source: Parasit Vectors. 2025 Dec 6;19:21. doi: 10.1186/s13071-025-07167-2 (PMC12797912; doi:10.1186/s13071-025-07167-2)
Supplement: Supplementary file 5 — Supplementary Material 5: Table S2. Double and multiple infections detected in cattle ticks in Lao People's Democratic Republic. [file 13071_2025_7167_MOESM5_ESM.docx]

Table S2. Double and multiple infections detected in cattle ticks in Lao People's Democratic Republic

| Location | Tick  species | Number of ticks | Number of double infections  (% of total positive prevalence) | | | | |  | Number of multiple infections  (% of total positive prevalence) | | |
| --- | --- | --- | --- | --- | --- | --- | --- | --- | --- | --- | --- |
|  |  |  | Am+  T | A+T | Bbo+  T | Am+  Bbi | Am+  Bbo | Bbo+  E | Am+Bbi  +T | E+Bbi  +T | Am+Bbi+  Bbo+T |
| LPB | *Rhipicephalus microplus* | 88 | - | - | - | - | - |  | - | - | - |
| KMN | *R. microplus* | 89 | 10  (11.2) | 2  (2.2) | 1  (1.1) | 2  (2.2) | - | 1  (1.1) | 4  (4.5) | 1  (1.1) | 1  (1.1) |
| CPS | *R. microplus* | 31 | - | - | - | 2  (6.4) | 2  (6.4) |  | - | - | - |
| CPS | *Rhipicephalus linnaei* | 19 | - | - | - | - | - |  | - | - | - |
| Total |  | 227 | 10  (4.4) | 2  (0.9) | 1  (0.4) | 4  (1.8) | 2  (0.9) | 1  (0.4) | 4  (1.8) | 1  (0.4) | 1  (0.4) |

Abbreviations: LPB: Luang Prabang, KMN: Khammouan, CPS: Champasak, A: *Anaplasma* sp., Am: *Anaplasma marginale,* Bbi: *Babesia bigemina*, Bbo: *Babesia bovis*, E: *Ehrlichia* sp*.*, T: *Theileria* sp.
